# IPCA-CMI: An Algorithm for Inferring Gene Regulatory Networks based on a Combination of PCA-CMI and MIT Score

**DOI:** 10.1371/journal.pone.0092600

**Published:** 2014-04-11

**Authors:** Rosa Aghdam, Mojtaba Ganjali, Changiz Eslahchi

**Affiliations:** 1 Department of Statistics, Faculty of Mathematical Sciences, Shahid Beheshti University, Tehran, Iran; 2 Department of Computer Science, Faculty of Mathematical Sciences, Shahid Beheshti University, Tehran, Iran; 3 School of Biological Science, Institute for Research in Fundamental Sciences (IPM), Tehran, Iran; Technische Universität Dresden, Medical Faculty, Germany

## Abstract

Inferring gene regulatory networks (GRNs) is a major issue in systems biology, which explicitly characterizes regulatory processes in the cell. The Path Consistency Algorithm based on Conditional Mutual Information (PCA-CMI) is a well-known method in this field. In this study, we introduce a new algorithm (IPCA-CMI) and apply it to a number of gene expression data sets in order to evaluate the accuracy of the algorithm to infer GRNs. The IPCA-CMI can be categorized as a hybrid method, using the PCA-CMI and Hill-Climbing algorithm (based on MIT score). The conditional dependence between variables is determined by the conditional mutual information test which can take into account both linear and nonlinear genes relations. IPCA-CMI uses a score and search method and defines a selected set of variables which is adjacent to one of 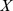 or *Y*. This set is used to determine the dependency between *X* and *Y*. This method is compared with the method of evaluating dependency by PCA-CMI in which the set of variables adjacent to both *X* and *Y*, is selected. The merits of the IPCA-CMI are evaluated by applying this algorithm to the DREAM3 Challenge data sets with *n* variables and *n* samples (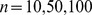) and to experimental data from *Escherichia coil* containing 9 variables and 9 samples. Results indicate that applying the IPCA-CMI improves the precision of learning the structure of the GRNs in comparison with that of the PCA-CMI.

## Introduction

Bayesian networks (BNs) provide an efficient and effective representation of the joint probability distribution of a set of variables. The identification of the structure of a BN from the data is known to be an NP-hard problem [1]. There are many learning algorithms for automatically building a BN from a data set. These are generally classified into three classes, namely constraint-based methods [2]–[5], score and search methods [6]–[11] and hybrid methods [12]–[14].

Gene Regulatory Networks (GRNs) explain how cells control the expression of genes. GRN is a collection of DNA segments in a cell. These segments interact indirectly with each other and with other substances in the cell and thereby governing the rates at which genes in the network are transcribed into Messenger RNA. Modeling the causal interactions between genes is an important and difficult task, and indeed, there are many heuristic methods for inferring GRNs from gene expression data [15], [16]. BN is one of the popular methods which have been successfully implemented in learning GRNs [17].

There is a great potential for improvement of current approaches for learning GRNs [18], [19]. The purpose of this study is to introduce a new algorithm, “Improved Path Consistency Algorithm based on Conditional Mutual Information (IPCA-CMI)”. The algorithm is applied to a number of gene expression data sets in order to evaluate the accuracy of it for inferring GRNs. IPCA-CMI is a combination of the PCA-CMI [5] and the Hill Climbing(HC) algorithm (based on mutual information test (MIT)) [11].

Being based on conditional mutual information (CMI), IPCA-CMI can take into account both linear and nonlinear genes relations. This is an improvement over linear testing methods. IPCA-CMI applies the HC algorithm (based on MIT score) to define weight values for each variable 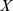. Then, a selected set which contains variables with weight values more than a defined threshold, is created. The method of evaluating dependency between two adjacent variables 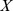 and 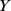 is represented by CMI test given a subset of genes of the selected set. To evaluate the accuracy of IPCA-CMI, it was employed to a number of gene expression data sets. For this purpose, the Dialogue for Reverse Engineering Assessments and Methods (DREAM) program was first introduced as a new efficient computation methods that help researchers to infer reliable GRNs [18]. The data sets comprised DREAM3 Challenge with 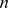 variables and 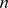 samples (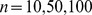) and *Escherichia coil* gene expression data containing 9 variables and 9 samples.

### Preliminaries

#### Bayesian network

Bayesian networks (BNs) [20], [21], also known as belief networks, belong to the family of probabilistic graphical models. Each vertex in the graph represents a random variable and the edges between the vertices represent probabilistic dependencies among the corresponding random variables. A directed edge, 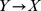, describes a parent and child relation in which 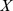 is the child and 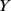 is the parent of 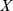. Let 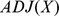 denotes the set of variables in the graph which are adjacent to 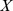. In addition, each vertex in graph has a conditional probability distribution specifying the probability of possible state of the variable given possible combination of states of its parents. These conditional dependencies in the graph are often estimated by using known statistical and computational methods. Hence, BNs combine principles from Graph Theory, Probability Theory, Computer Science and Statistics. BNs are represented as a directed acyclic graph (DAG) that is popular in Statistics and Machine Learning subjects. We typically denote random variables with capital letters and sets of random variables as bold capital letters. Following the above discussion, a more formal definition of a BN can be given. A Bayesian network, 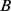, is an annotated directed acyclic graph that represents a joint probability distribution over a set of random variables 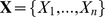. The network is defined by a pair 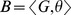, where 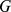 is the DAG with vertex set 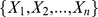 and the direct dependencies between these variables is represented by directed edges. The graph 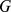 encodes independence assumptions, by which each variable 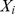 is independent of its non descendants given its parents in 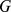. Let 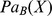 denote the parent set of 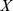. The second component 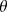 describes the set of conditional probability distributions. This set contains the parameter 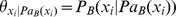, where 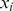 denotes some value of the 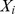 and 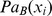 indicates some set of values for 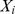
's parents. If 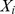 has no parent, then 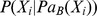 is equal to 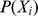. By using these conditional distributions, the joint distribution over 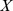 can be obtained as follows:
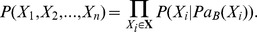



**Definition 1**. If 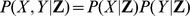, then two variables 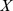 and 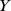 are conditionally independent given 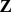.

**Definition 2**. A path 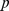 from 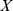 to 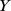 in 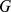 is said to be blocked by a set of variables 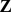 if and only if:

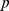
 contains a chain X
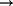
K
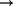
Y or a fork X
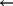
K
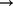
Y such that 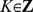, or

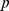
 contains a collider X
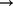
K
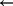
Y such that K and all the descendants of K are not in 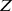.

**Definition 3**. A set 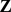 is said to d-separate 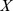 from 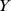 in 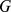 if and only if 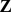 blocks every path from 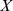 to 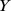.

**Definition 4**. A v-structure in 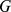 is an ordered triplet 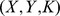 such that 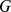 contains the directed edges X
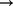
Y and K
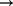
Y, so that 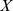 and 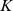 are not adjacent in 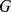.

For the following discussion, suppose that the set of parents of 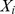 is 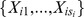, where 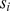 denotes the number of parents of 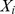
(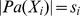). The BN deals with:

- Discrete variables i.e. the variable 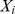 and its parents take discrete values from a finite set. Then, 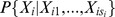 is represented by a table that specifies the probability of values for 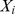 for each joint assignment to 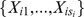.
- Continuous variables i.e. the variable 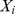 and its parents take real values. In this case, there is no way to represent all possible densities. A natural choice for multivariate continuous distributions is the use of Gaussian distributions [15].
- Hybrid networks i.e. the network contains a mixture of discrete and continuous variables.

#### Information Theory

Gene expression data are typically modeled as continuous variables. The following steps are applied to calculate mutual information (MI) and CMI for continuous variables. MI has been widely used to infer GRNs because it provides a natural generalization of association due to its capability of characterizing nonlinear dependency [22]. Furthermore, MI is able to deal with thousands of genes in the presence of a limited number of samples [23].

Entropy function is a suitable tool for measuring the average uncertainty of a variable 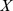. Let 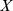 be a continuous random variable with probability density function 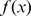, the entropy for 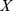 is:
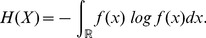
(1)The joint entropy for two continuous variables 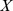 and 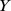 with joint density function 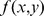 is:
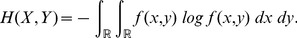
(2)The measure of MI indicates the dependency between two continuous variables 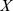 and 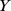, which is defined as:
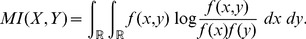
(3)Variables 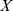 and 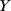 are independent when MI has zero value. The measure of MI can also be determined in terms of entropy as follows:
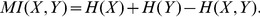
(4)In the GRN the dependency of two genes needs to be determined. CMI is a suitable tool for detecting the joint conditional linear and nonlinear dependency between genes [5], [24]. CMI between two variables 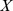 and 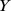, given the vector of variables 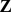 is:
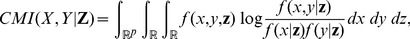
(5)where 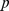 is the dimension of vector **Z** and 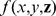 denotes the joint density function for variables and 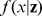 is the conditional density distribution of 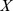 given 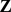. CMI between 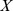 and 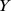 given 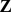 can also be expressed by:
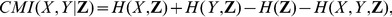
(6)where 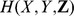 denotes the joint entropy between 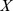, 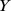 and 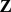.

**Theorem 1** [25]: Let 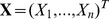 be an 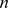-dimensional Gaussian vector with mean 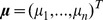 and covariance matrix 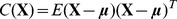, i.e. 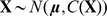. The entropy of 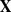 is:
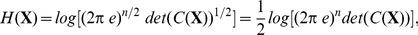
(7)where 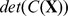 indicates the determinant of 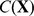. With the widely adopted hypothesis of Gaussian distribution for gene expression data, the measure of MI according to Eqs. 4 and 7 for two continuous variables 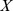 and 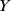 can be easily calculated using the following equivalent formula [5], [26]:
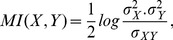
(8)where 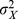, 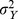 and 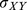 indicate the variance of 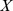, the variance of 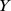 and the covariance between 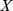 and 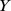. Similarly, according to Eqs. 6 and 7, CMI for continuous variables 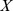 and 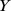 given 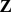 can be determined by [5]:
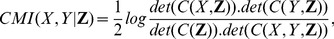
(9)in which 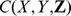 denotes the covariance matrix of variables 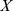, 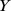 and 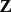. When 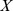 and 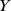 are conditionally independent given 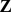, then 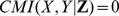. In order to test whether a CMI is zero, 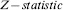 is calculated in two steps [5], [27], [28]:

In step 1, the MI and CMI, respectively, are normalized as follows:
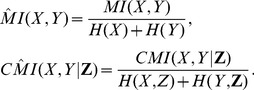
(10)In step 2, the 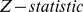 of MI and CMI, respectively, are calculated by:
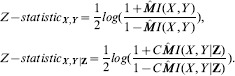
(11)In order to determine the statistical test of conditional independence, a confidence level 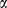 is fixed. When 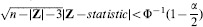 then, the hypothesis of conditional independence of 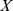 and 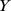 given 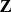 is accepted (at the significance level 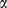); otherwise the hypothesis is rejected. Here 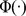 denotes the cumulative distribution function of the standard normal distribution and 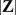 indicates the dimension of vector **Z**.

### Score and Search Algorithms

Score and search algorithms can be completely described by specifying two components: a scoring function and a search procedure. The score and search algorithms try to identify a network with maximum score.

In this study, we apply the HC algorithm as a search procedure where MIT score is used as a scoring function. Gene expression data are typically continuous variables. The MIT score deals with discrete variables. Therefore, continuous variables have to be discretized. We do this based on the procedure proposed by [29]–[32].

#### Discretization methods

To draw inferences about a GRN based on the set of genes 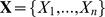, we start with a data set 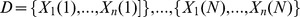, where 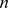 indicates the number of genes and 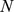 is the number of measurements of these genes. An 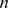 by 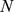 matrix 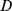 is used to denote gene expression data. 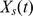 indicates the expression value of gene 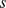 at time 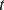. 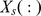 denotes expression data of gene 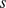 at all time. The equal width discretization (EWD) and equal frequency discretization (EFD) methods are applied to discretize continuous gene expression data [30]–[32]. EWD method for 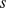-th gene divides the line between 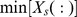 and 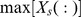 into 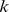 intervals of equal width. Thus the intervals of gene 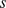 have width, 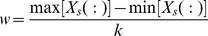, with cut points at 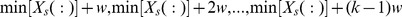. In EWD, 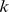 is a positive integer and is a user predefined parameter.

EFD method for 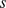-th gene divides the sorted 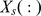 into 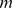 intervals so that each interval contains approximately the same number of expression values. Similarly, in EFD, 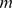 is a positive integer and is a user predefined parameter.

In this study, gene expression data sets related to DREAM3 Challenge lie in the interval [0, 1]. We applied EWD method to discretize DREAM3 data sets. For instance, for each gene, parameter 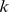 is considered to be equal to 10. EFD method is applied to discretize SOS repair data. Gene expression data sets related to SOS DNA repair network lie in the interval [−0.2730, 26.6330] and the parameter 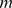 is considered to be equal to 9.

### Scoring Function

There are many scoring functions to measure the degree of fitness of a DAG 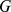 to a data set. These are generally classified as Bayesian scoring functions [7], [9], [33] and information theory-based scores [11], [34]–[37]. The chosen score and search algorithm can be more efficient if the scoring function has the decomposability property.

**Decomposability property**: A scoring function 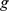 is decomposable if:
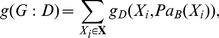
(12)where
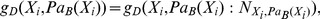
(13)and 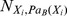 denotes the number of instances in data set 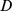 that match with each possible configuration of 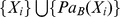.

Another property, which is particularly interesting if the score and search algorithm searches in a space of equivalence classes of DAGs, is called the score equivalence.

**Theorem 2** [38]. Two DAGs are equivalent if and only if they have the same skeletons and the same v-structures.

When two Bayesian networks are equivalent, they can represent the same set of probability distributions. The relation of network equivalence imposes a set of equivalence classes over Bayesian network structures [39].

**Score equivalence**: A scoring function 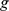 is score equivalence if the score assigns the same value to equivalent structures.

#### MIT Score

Mutual information test (MIT) belongs to the family of information theory-based scores which is defined as follows [11]:
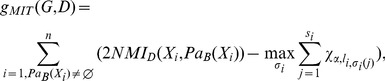
(14)where N denotes the total number of measurements in 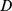 and 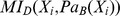 is determined by:
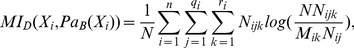
(15)where 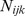 represent the number of measurements in the data set 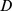 for which 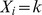 and 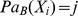, where 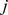 denotes a joint configuration of all parent variables of 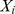. 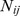 denotes the number of measurements in 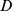, in which 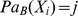. Similarly, 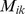 indicates the number of measurements in 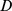 which the variable 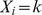 and 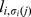 is defined by:
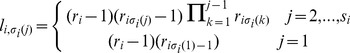
(16)where 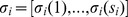 indicates any permutation of the index set 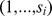 of the variables in 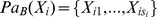. Finally, 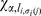 is the value such that 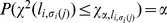 (the Chi-squared distribution at significance level 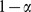 with 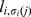 degrees of freedom).

The MIT score has decomposability property and dose not satisfy score equivalence, however, it satisfies less demanding property. This property of the MIT score concerns another type of space of equivalent of DAGs, namely restricted acyclic partially directed graphs (RPDAGs) [40]. RPDAGs are partially directed acyclic graphs (PDAGs) which represent sets of equivalent DAGs, although they are not a canonical representation of equivalence classes of DAGs (two different RPDAGs may correspond to the same equivalence class).

**Theorem 3** [11]. The MIT score assigns the same value to all DAGs that are represented by the same RPDAG.

MIT score can be applied without any problem to search in both the DAG and the RPDAG spaces [11]. In different studies, the score equivalence could be concluded as a good or bad property. The score equivalence property is appropriate when the data are not applied to distinguish the equivalent structures. In searching and scoring scheme for learning structure of Bayesian networks, equivalent classes should be considered. This means when more than two graphs are equivalent, those graphs have the same dependency; therefore, two structures have identical scores. As an example, two variables *A* and *B* may have two different structures as 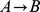 or 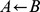, however, as equivalent classes, these two structures end up having the same score for any given data. In order to detect causal relationships between genes, score equivalence property does not necessarily impair the search process, because equivalent structures represent different causal relationships. In this study, we are interested to the scoring functions which considered different scores for 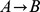 or 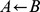. So, MIT score is applied in the HC algorithm to compute the score of DAGs. The non equivalence of the score function does not necessarily impair the search process to learn BNs. The MIT score is implemented within the Elvira system (a JAVA package for learning the structure of BN [11]). The Elvira package can be downloaded from http://leo.ugr.es/elvira/. The MIT score is available at 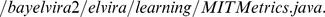 In this study, we rewrite the MIT score program (Red.Pen) which, in comparison to the Elvira system, reduces running time and memory occupied by the algorithm. The source of the program and data sets are available at http://www.bioinf.cs.ipm.ir/software/IPCA-CMI/.

### Search Procedure

Given a scoring function 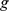, the task in this step relates to search between possible networks to find 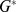 such that:
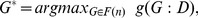
(17)in which 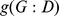 denotes the degree of fitness of candidate 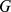 to data set and 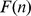 indicates all the possible DAGs defined on 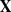. The challenging part of search procedure is that the size of the space of all structures, 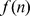, is super-exponential in the number of variables [41],
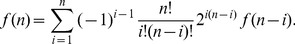
(18)So an exhaustive enumeration of all the structures is not possible. Instead, researchers have considered heuristic search strategies [9], [42]. The Hill Climbing algorithm is particularly popular in this field.

#### The Hill Climbing Algorithm

The Hill Climbing (HC) algorithm is a mathematical optimization technique which belongs to the family of local search. The HC algorithm traverses the search space by starting from an initial DAG then, an iterative procedure is repeated. At each procedure, only local changes such as adding, deleting or reversing an edge are considered and the greatest improvement of *g* is chosen. The algorithm stops when there is no local change yielding an improvement in *g*.

Because of this greedy behavior the execution stops when the algorithm is trapped in a solution that is mostly local rather than global maximizer of *g*. Different methods are introduced to escape from local optima such as restarting the search process with different initial DAGs. It means that after a local optima is found the search is reinitialized with a random structure. This reinitialization is then repeated for a fixed number of iterations, and the best structure is selected [20]. The local search methods can be more efficient if the scoring function has the decomposability property [11]. By considering the decomposability property, by adding, deleting or reversing the edge between two variables, the score values of this variables are updated while the score values of other variables remain unchanged. In order to apply the HC algorithm based on scoring function with the decomposability property, the following differences are calculated to evaluate the improvement obtained by local change in a DAG [43]:

1. Addition of 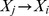: 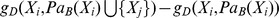

2. Deletion of 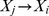: 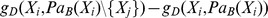

3. Reversal of 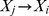: First the edge from 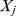 to 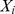 is deleted then, a edge from 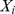 to 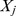 is added. So 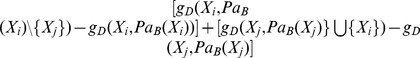 is computed.

## Method

In this section the details of PCA-CMI and IPCA-CMI are presented to show how the structure of GRN is learned from gene expression data sets.

### PC Algorithm based on CMI test (PCA-CMI)

The PCA-CMI is applied to infer the GRNs [5]. The PCA-CMI is computationally feasible and often runs very fast on networks with many variables. This algorithm starts with a complete undirected graph over all variables. The following steps are applied to assign skeleton 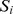 from 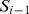.

Step 1: Generate the complete undirected graph 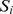 (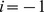).

Step 2: Set 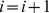. Suppose 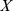 and 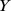 are adjacent in 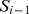, then 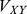 is defined by:
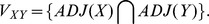
Suppose that, there are 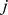 number of genes in 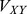 (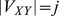). If 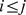, for each *i*-subset of 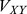 such as 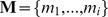, the *i*-order 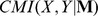 is computed according to Eq. 9. All the *i*-order CMIs between *X* and *Y* given all possible combination of *i* genes from *j* genes are computed and the maximum one was selected as 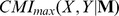. If 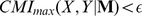, the edge between *X* and *Y* is removed from 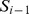. So, 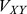 includes the separator set for *X* and *Y*. The algorithm is stopped when 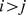. Let 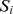 be the skeleton of the constructed graph in this step and return to step 2.

The algorithm is stopped when 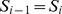 for the first *i*.

It is notable that in each step of the PCA-CMI, 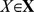 is selected from 1 to *n* and 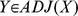 is selected by the order of genes. Details of PCA-CMI are given in table 1.

**Table 1:** The PC Algorithm based on CMI test (PCA-CMI) [5].

1:	Start with a complete undirected graph 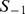
2:	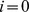
3:	Repeat
4:	For each 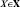
5:	For each 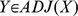
6:	Determine if there is 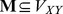 with 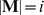 such that 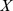 and 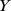 given **M** are independent
7:	If this set exists
8:	Remove the edge between *X* and *Y* from 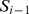
9:	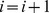
10:	Until 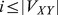

### The Improvement of PC Algorithm based on CMI test (IPCA-CMI)

The HC algorithm is the well-known approach to search between possible DAGs to determine the best fit of network based on defined scoring function. In addition, Zhang [5] have implemented the PCA-CMI for inferring GRNs from gene expression data, using CMI test in the process of dependency determination between genes. The skeleton of a GRN in each order of IPCA-CMI is determined by CMI test. Therefore, only the local changes related to reversed edges between genes are applied in the HC algorithm (step 3 of the algorithm) in order to direct the edges of the skeleton.

When heuristic search algorithms are applied, we are not guaranteed to find a global optima structure. Different methods have been proposed to escape local optima.

In this study, during each iteration in the HC algorithm, a new solution is selected from the neighborhood of the current solution (random change including adding, deleting and reversing). If that new solution has a better quality MIT score, then the new solution becomes the current solution. The algorithm stops if no further improvement are possible. We have to start with some (50 randomly generated) solution and evaluate it based on MIT score. The HC algorithm can only provide locally optima that depends on the starting solution. We have to start the HC algorithm from a large variety of different solutions. The hope is that at least some of these initial locations have a path that leads to the global optima. We choose the initial solutions (50 DAGs) at random.

Details of the IPCA-CMI are presented in two parts. Part 1 is related to the zero order of the IPCA-CMI. In this order, same skeletons are obtained by PCA-CMI and IPCA-CMI, but the HC algorithm is utilized in IPCA-CMI in order to direct the edges of the skeleton. Details of the IPCA-CMI for order *i* (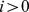) are presented in part 2.

#### Part 1: The details of IPCA-CMI for 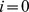


First, the IPCA-CMI generates complete graph according to the number of genes. Then, for each adjacent gene pair such as *X* and *Y*, the measure of MI is computed according to equation [8]. The measures of MI between *X* and *Y* are calculated to be compared with 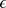. If 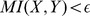, the edge between *X* and *Y* is removed from complete graph. Finally, MIT score is applied in the HC algorithm in order to direct the edges of skeleton to obtain the directed acyclic graph 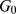. Details of zero order of IPCA-CMI are shown in table 2.

**Table 2:** Zero order of the Improvement of PC Algorithm based on CMI test.

1:	Start with a complete undirected graph 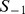 .
2:	Repeat
3:	For each 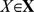
4:	For each 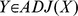
5:	If *X* and *Y* are independent based on the measure of MI
6:	Remove the edge between *X* and *Y* from 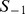
7:	The MIT score was utilized in the HC algorithm to construct 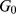 .

#### Part 2: The IPCA-CMI for 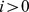


Set 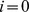 and the following process is applied to assign directed acyclic graph 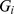 from 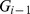:

Step 1: Set 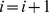. Let 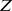 be an adjacent of 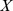 in 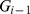. Then, 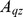 for 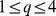 are defined as follows:
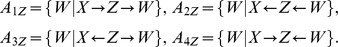
The weight value for variable *Z* is determined by:
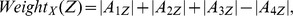
where 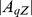 for 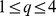 denotes the size of 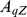.

Step 2: Let 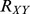 be defined by:
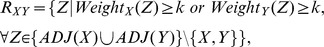
where *k* denotes the median of weights related to all adjacent variables of *X* or *Y*. It can be concluded that variables in set 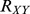 are selected from 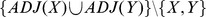 in which at least *k* number of paths started from *X* or *Y* are blocked by these variables. Therefore, by considering these variables many paths between *X* and *Y* are removed.

Step 3: Let *X* and *Y* be adjacent in 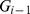, we have done the following process:

Suppose that, there are *t* genes in 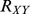 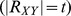. If 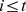, for each 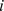-subset of 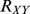 such as 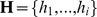, the *i*-order 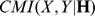 is computed according to equation [9]. All the 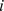-order CMIs between *X* and *Y* given all possible combination of *i* genes from *t* genes are computed and the maximum result (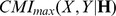) is compared with 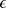. If 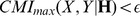, the edge between X and Y is removed from 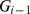. The algorithm is stopped when 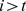. Let 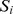 be the skeleton of the constructed graph in this step.

Step 4: MIT score is applied in the HC algorithm in order to direct the edges of 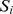 to obtain the directed acyclic graph 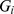, return to step 1.

The algorithm is stopped when 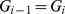 for the first *i*.Table 3 is related to the details of i order (i>0) of IPCA-CMI.

**Table 3:** *i* order (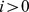) of the Improvement of PC Algorithm based on CMI test.

1:	Start with 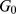
2:	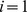
3:	Repeat
4:	For each 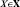
5:	For each 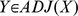
6:	Test whether 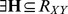 with 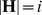 such that *X* and *Y* given **H** are independent.
7:	If this set exists
8:	Remove the edge between *X* and *Y* from 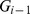
9:	The MIT score was utilized in the HC algorithm to direct the structure.
10:	For each 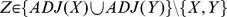
11:	The weight value for variable 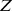 is determined by: 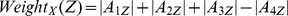
12:	A selected set 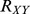 of variables is created as: 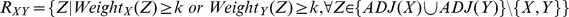
13:	i = i+1
14:	Until 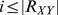

It is notable that in each step of the IPCA-CMI, 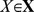 is selected from 1 to n and 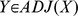 is selected by the order of genes.

The rational behind 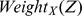 is in definitions 2 and 3. 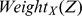 indicates the number of paths started from 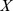 and blocked by 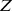.

In fact the main difference between the IPCA-CMI and the PCA-CMI is in choosing a selected set of variables which includes the separator set. IPCA-CMI uses the HC algorithm and define a selected set of variables which are adjacent to one of *X* or *Y*, with weight values more than a defined threshold.

### Software

Software in the form of MATLAB and JAVA codes. The source of data sets and codes are available at http://www.bioinf.cs.ipm.ir/software/IPCA-CMI/.

## Results

In order to validate our algorithm, the true positive (TP), false positive (FP), true negative (TN) and false negative (FN) values for proposed algorithms are computed. Where TP is the number of edges that are correctly identified, FP is the number of edges that are incorrectly identified, TN is the number of edges that are correctly unidentified and FN is the number of edges that are incorrectly unidentified. In addition, some famous measures such as the accuracy (ACC), false positive rate (FPR), false discovery rate (FDR), positive predictive value (PPV), F-score measure, Matthews correlation coefficient (MCC) and true positive rate (TPR) are considered to compare algorithms, more precisely. These measures are defined by:
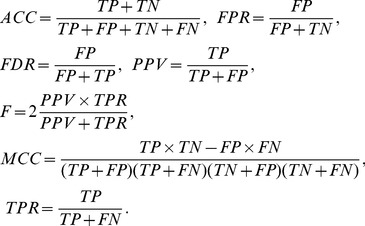
(19)MCC is a convenient quantity for comparing predicted and actual networks. MCC quantity for each algorithm indicates which method is more efficient in predicting networks. The algorithm which has higher values for measures TP, TN, ACC, PPV, F, MCC and TPR is more efficient for predicting the skeleton of networks.

The DREAM3 Challenge consists of 4 data sets that were produced from in-silico networks. The goal of the in-silico Challenge is the reverse engineering of gene networks from time series data. The gold standard for DREAM3 Challenge were determined from source networks of real species. In this study, we tested the performance of IPCA-CMI on the DREAM3 data sets with *n* variables and *n* samples (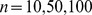) and to experimental data from *Escherichia coil* containing 9 variables and 9 samples. Data sets contain the expression values of genes, in which rows are genes and columns indicate the samples. In order to compare results of PCA-CMI and IPCA-CMI, in each algorithm, we used the same threshold for CMI tests previously applied by Zhang [5].

IPCA-CMI, is a combination of a constraint-based method named PCA-CMI with a score and search method named the HC algorithm. Since the HC algorithm includes the process of randomly selecting initial graphs, IPCA-CMI is supposed to run hundred times and then we take the average as the final result. It can be concluded that outcomes of Tables 1 to 5 are related to the average of results which obtained from IPCA-CMI in hundred times.

**Table 4:** The result of Simulated and Real data sets in order 0.

Network	TP	FP	ACC	FPR	FDR	PPV	F	MCC	TPR
DREAM10	9	1	0.95	0.02	0.10	0.9	0.90	0.87	0.90
DREAM50	36	54	0.92	0.05	0.6	0.4	0.43	0.39	0.46
DREAM100	70	58	0.96	0.01	0.45	0.55	0.47	0.46	0.42
SOS	18	4	0.72	0.33	0.18	0.82	0.78	0.40	0.75

The second row of the table shows the result of DREAM3 in size of 10 with threshold 0.05. The third row denotes the result of DREAM3 in size of 50 with threshold 0.1. The forth row of the table indicates the result of DREAM3 in size of 100 with threshold 0.1. Finally the last row shows the result of SOS DNA repair network with threshold 0.01.

**Table 5:** The result of gene expression data set DREAM3 Challenge with 10 genes and sample number 10.

Algorithm	TP	FP	ACC	FPR	FDR	PPV	F	MCC	TPR
PCA1	7	1	0.91	0.03	0.13	0.87	0.78	0.73	0.7
IPCA1	**8.8**	**0**	**0.98**	**0**	**0**	**1**	**0.94**	**0.93**	**0.88**

Result of DREAM3 in size of 10 with first-order CMI test with threshold 0.05. The second row of the table indicates the result of first-order PCA-CMI (PCA1) the third row of the table shows the result of first-order IPCA-CMI (IPCA1).

**Figure 1:**
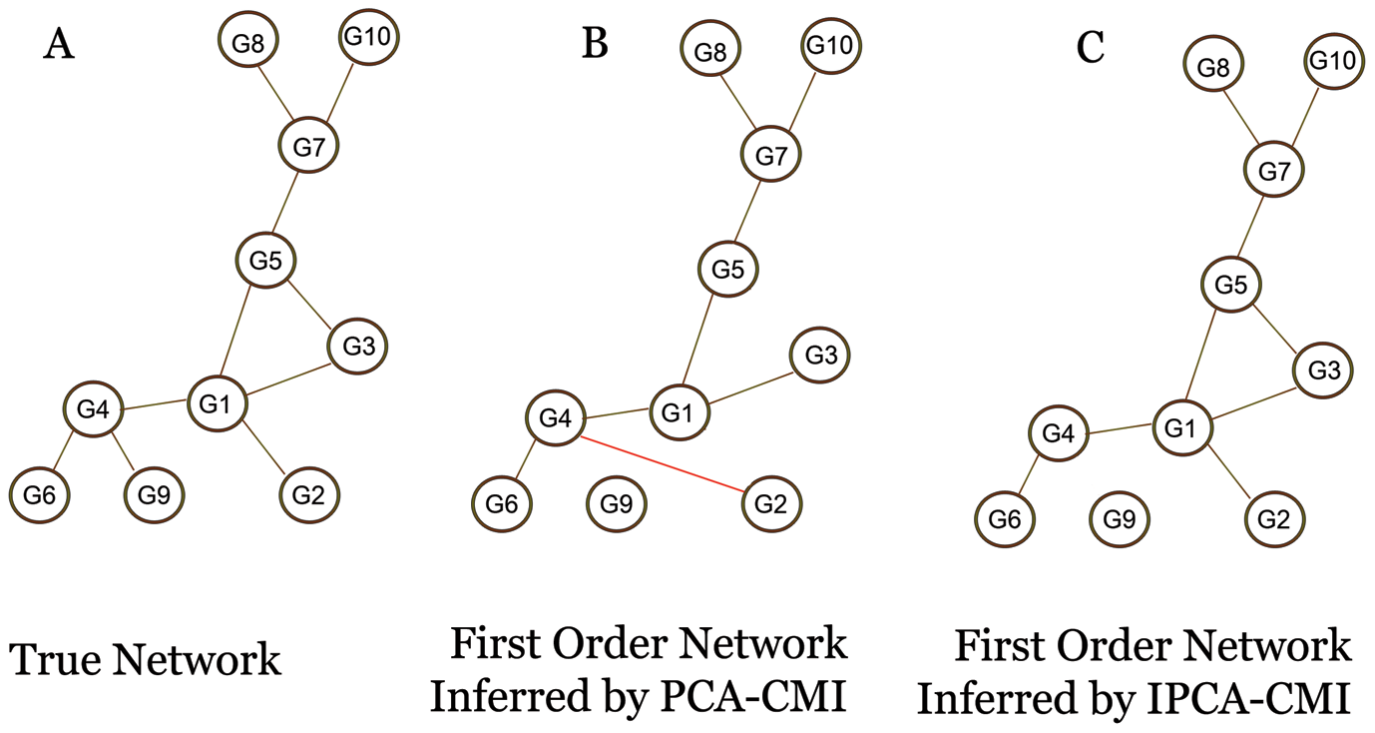
Comparing the result of the PCA-CMI and the IPCA-CMI for inferring the structure of DREAM3 contains 10 variables and 10 edges. (A) The true network with 10 variables and 10 edges. (B) Firs-order network inferred by the PCA-CMI. The edge with red line G2–G4 is false positives, while the edges G1–G2, G3–G5 and G4–G9 are false negative. (C) First-order network obtained by the IPCA-CMI. The false positive edge G2–G4 in (B) is successfully removed by the IPCA-CMI, in addition edges G1–G2 and G3–G5 are successfully found by this algorithm.

Fig. 1(A) shows the structure of true network for DREAM3 which contains 10 genes and 10 edges. The result obtained by Zhang [5] illustrated in Fig. 1(B), and Fig. 1(C) is related to the result of IPCA-CMI. In Fig. 1(B), edges that are correctly found by PCA-CMI are shown in Black color and the edge that wrongly inferred by this algorithm (edge G2–G4) is shown in red color. The true edges, which found by IPCA-CMI, are indicated by Black color and edge G4–G9 is a false negative. Fig. 1(C) is related to the best result of IPCA-CMI in running it hundred times.

Table 4 indicates the result of PCA-CMI and IPCA-CMI with zero-order CMI test for DREAM3 and SOS real gene expression data. In zero-order two algorithms returned the same results, since both algorithms contain the same procedure.

Table 5 indicates the result of PCA-CMI and IPCA-CMI for DREAM3 data set in size of 10 genes with 10 edges. We set the threshold value 0.05 of MI and CMI tests for dependency determination. As shown by Table 5, TP, ACC, PPV, F, MCC and TPR under PCA-CMI are less than those of IPCA-CMI. So, it can be concluded that the IPCA-CMI is more suitable for structure learning.

Results of applying PCA-CMI and IPCA-CMI for DREAM3 Challenge with 50 genes and 77 edges are collected in Table 6. We chose 0.1 as the threshold value of MI and CMI tests to determine the dependency between genes. IPCA-CMI can detect the true network in 2 steps, FP value is reduced as a result of applying algorithm step by step. According to Table 6 the FP value is reduced from 21 to 11.78, as a result of using IPCA-CMI. The TP, ACC, PPV, F, MCC and TPR measures receive higher values by using IPCA-CMI for inferring GRNs which shows that the IPCA-CMI performs better than the PCA-CMI.

**Table 6:** The result of gene expression data set DREAM3 Challenge with 50 genes and sample number 50.

Algorithm	TP	FP	ACC	FPR	FDR	PPV	F	MCC	TPR
PCA1	24	23	0.93	0.02	0.49	0.51	0.39	0.37	0.31
PCA2	22	21	0.93	0.02	0.49	0.51	0.37	0.35	0.29
IPCA1	**28**	26.5	**0.94**	0.02	**0.48**	0.51	**0.43**	**0.4**	**0.36**
IPCA2	**22.9**	**11.78**	**0.95**	**0.01**	**0.48**	**0.52**	**0.38**	**0.42**	**0.3**

Result of DREAM3 in size of 50 with different CMI orders with threshold 0.1. The second and third rows of the table indicate the result of first-order PCA-CMI (PCA1) and second-order PCA-CMI (PCA2), respectively. The forth and fifth rows of the table show the result of IPCA-CMI of first-order(IPCA1) and second-order(IPCA2), respectively.

Results of DREAM3 with 100 variables and 166 edges are illustrated in Table 7. Threshold value 0.1 for MI and CMI tests is considered to determine the dependency between genes. As shown by Table 7 in the second-order network, the FP value is reduced from 25 to 15.16. The TP, ACC, PPV, F, MCC and TPR measures receive higher values by using IPCA-CMI for inferring about DREAM3 with 100 variables. Results of applying PCA-CMI and IPCA-CMI for the real data set with 9 genes and 24 edges are given in Table 8. We chose 0.01 as the threshold value. Table 8 indicates that ACC, F and MCC measures receive higher values by using IPCA-CMI for inferring about BNs which shows that the IPCA-CMI performs better than the PCA-CMI.

**Table 7:** The result of gene expression data set DREAM3 Challenge with 100 genes and sample number 100.

Algorithm	TP	FP	ACC	FPR	FDR	PPV	F	MCC	TPR
PCA1	49	25	0.971	0.005	0.34	0.66	0.41	0.43	0.28
PCA2	46	25	0.971	0.005	0.35	0.64	0.38	0.41	0.27
IPCA1	**53.11**	29.77	**0.972**	0.006	0.35	0.65	**0.43**	**0.44**	**0.32**
IPCA2	**46.55**	**15.16**	**0.973**	**0.003**	**0.24**	**0.75**	**0.4**	**0.45**	**0.28**

Result of DREAM3 in size of 100 with different CMI orders with threshold 0.1. The second and third rows of the table indicate the result of first-order PCA-CMI (PCA1) and second-order PCA-CMI (PCA2), respectively. The forth and fifth rows of the table show the result of IPCA-CMI of first-order (IPCA1) and second-order (IPCA2), respectively.

**Table 8:** The result of experimental data from *Escherichia coil* containing 9 genes and sample number 9.

Algorithm	TP	FP	ACC	FPR	FDR	PPV	F	MCC	TPR
PCA1	18	4	0.72	0.33	0.18	0.82	0.78	0.40	0.75
IPCA1	**18**	**1.8**	**0.73**	**0.32**	**0.17**	0.82	**0.79**	**0.41**	0.75

The result of SOS DNA repair network in size of 9 with 24 edges. Results are related to the order 1 of CMI with threshold 0.01. The second row of the table indicates the result of first-order PCA-CMI (PCA1). The third row of the table show the result of IPCA-CMI of first-order (IPCA1).

According to Tables 4 to 8, the number of FP is decreased, as a result of using IPCA-CMI. So it can be concluded that the IPCA-CMI is more suitable for learning the structure of GRNs. Tables (4 to 8) show that IPCA-CMI not only can reduce the number of FP but also it remarkably can find some true different edges in comparison with PCA-CMI. As shown by these Tables (4 to 8), some better results can be obtained by using IPCA-CMI. So, it can be concluded that IPCA-CMI performs better than the PCA-CMI for learning the structure of GRNs. Another comparison that can be made between these algorithms is a determination of the probability of selecting subgraph with *k* edges from graph *G* with *m* edges. These probabilities are calculated for two mentioned algorithms. The algorithm which receives smaller value of the probability is efficient for predicting the skeleton of GRN. Results of this comparison for networks which are obtained using DREAM3 and SOS real gene expression data are given in Table 9. As shown by Table 9, better results (e.g., smaller probability values) are obtained by using IPCA-CMI. Therefore, it can be concluded that the performance of IPCA-CMI is much better than that of PCA-CMI based on the better determination of the probability for selecting a subgraph in all data sets.

**Table 9:** The probability of occurrence of GRNs.

Algorithm	DREAM10	DREAM50	DREAM100	SOS
PCA	1.948475e-05	6.211307e-17	9.751598e-53	0.01755
IPCA	**1.12846e-08**	**1.252285e-21**	**5.337701e-59**	**0.001215584**

A determination of the probability of selecting a subgraph using the PCA-CMI and the IPCA-CMI. The second row of the table indicates the result of last order PCA-CMI (PCA). The third row of the table show the result of last order IPCA-CMI (IPCA).

## Discussion

In this study a new algorithm called IPCA-CMI for inferring GRNs from gene expression data was presented. Results of this study show that using IPCA-CMI improves the precision of the learning the structure of GRNs, considerably. Zhang [5] reported that the PCA-CMI performed better than linear programming method [44], multiple linear regression Lasso method [45], mutual information method [46] and PC-Algorithm based on partial correlation coefficient [27] for inferring networks from gene expression data such as DREAM3 Challenge and SOS DNA repair network. Therefore, it can be concluded that the results of IPCA-CMI will be more precise compared to the methods studied by Zhang [5].

Our algorithm starts with a complete undirected graph over all variables. IPCA-CMI constructs 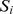 (the skeleton of order *i*) according to CMI test. Then perform the HC algorithm to direct the edges of 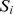. If *X* and *Y* are adjacent in 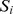, weight values are defined for variables in set 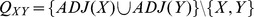. Subsequently, variables with high weight values were selected as the members of the set 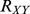. The separator set being a subset of 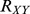, makes defining the set 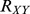 in the algorithm very important. We adopted a method to select *i* number of genes from 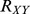. Suppose that, there are *t* number of genes in 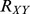 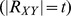. In order to construct the *i*-order 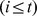 network, all the *i*-order CMIs between *X* and *Y* given all possible combination of *i* genes from *t* genes are calculated and the maximum result compared with 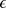 threshold to decide whether to keep the edge between 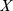 and 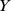 or to remove it.

The PC algorithm starts with a complete undirected graph over all variables. In order to construct 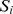, the Chi-square test is applied to determine dependency between variables. The separator set for adjacent genes 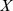 and 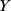 in 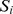 are selected from 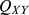. The PC algorithm is fast to learn networks with many variables. The drawback of the PC algorithm is the requirement for large sample sizes to perform high order conditional independence (CI). The number of records in a microarray data set is rarely sufficient to perform reliable high-order CI tests. Using IPCA-CMI statistical error in the process of learning the skeleton of GRNs is reduced. This is a result of the reduction of the size of the set which includes the separator set.

On the other hand in PCA-CMI, only genes connected with both *X* and *Y* are considered for dependency determination. It means that the separator set for two adjacent genes *X* and *Y* are selected from 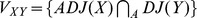. So, small set of variables are considered for dependency determination. It can be concluded that some of the variables which play an important role in dependency determination are not considered in separator set. The achieved improvement of our algorithm in comparison with PCA-CMI is related to the consideration of important adjacent genes of one of *X* or *Y*. This method leads us to determine the separator set for *X* and *Y* more precisely.

For the aforementioned problem for PC and PCA-CMI, in this study we applied an iterative strategy to select 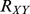 which includes separator set for adjacent genes *X* and *Y*. It can be concluded that 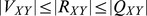. It means that, we chose the set of variables, among which to pick the separator set, in a somehow intermediate way between the standard PC algorithm and the method of Zhang et al. (2012). Therefore, the set of variables, among which we pick the separator set, is bigger than those considered by Zhang et al. (2012). The MIT scoring function is decomposable and is not score equivalent. However, it satisfies a restricted form of score equivalence which allows us to use it to search not only in the DAG space but also in the RPDAG space. In the future work we would like to investigate whether MIT score is more appropriate for gene expression data than other scores. It has been previously shown that the score equivalence is not an important feature to learn Bayesian networks by searching in the DAG space. This confirms the previous results stated by [11], [47]. Gene expression data are typically modeled as continuous random variables. The MIT score can be applied in analyzing continuous random variables, but only after the data has been discretized. In the future work we would like to apply a more suitable method to discretize gene expression data [29].
